# *MuTAnT*: a family of *Mutator*-like transposable elements targeting TA microsatellites in *Medicago truncatula*

**DOI:** 10.1007/s10709-015-9842-5

**Published:** 2015-05-17

**Authors:** Krzysztof Stawujak, Michał Startek, Anna Gambin, Dariusz Grzebelus

**Affiliations:** Institute of Plant Biology and Biotechnology, University of Agriculture in Krakow, Al. 29 Listopada 54, 31-425 Kraków, Poland; Institute of Informatics, University of Warsaw, Warsaw, Poland; Mossakowski Medical Research Centre, Polish Academy of Sciences, Warsaw, Poland

**Keywords:** Barrel medic, DNA transposon, MULE, Simple sequence repeats

## Abstract

Transposable elements (TEs) are mobile DNA segments, abundant and dynamic in plant genomes. Because their mobility can be potentially deleterious to the host, a variety of mechanisms evolved limiting that negative impact, one of them being preference for a specific target insertion site. Here, we describe a family of *Mutator*-like DNA transposons in *Medicago truncatula* targeting TA microsatellites. We identified 218 copies of *MuTAnTs* and an element carrying a complete ORF encoding a *mudrA*-like transposase. Most insertion sites are flanked by a variable number of TA tandem repeats, indicating that *MuTAnTs* are specifically targeting TA microsatellites. Other TE families flanked by TA repeats (e.g. *TAFT* elements in maize) were described previously, however we identified the first putative autonomous element sharing that characteristics with a related group of short non-autonomous transposons.

**Electronic supplementary material:**

The online version of this article (doi:10.1007/s10709-015-9842-5) contains supplementary material, which is available to authorized users.

## Introduction

Transposable elements (TEs) are mobile DNA segments present in most organisms. In higher plants, their content varies from 10 % in *Arabidopsis* (Arabidopsis Genome Initiative 2000) to more than 80 % in maize (Schnable et al. 2009). With respect to the transposition mechanism, TEs are divided into two classes; class I (retrotransposons) transpose via an RNA intermediate while class II (DNA transposons) change their location by a cut-and-paste mechanism characteristic for TEs carrying terminal inverted repeats (TIRs) or a rolling-circle mechanism typical for *Helitrons* (Wicker et al. 2007). A family of DNA transposons usually consists of one or a few autonomous elements capable of inducing their own transposition and more copies with internal deletions and rearrangements, referred to as non-autonomous, which lost the ability to transpose independently, however, they can be mobilized by a related autonomous element (Wessler 2006).

The canonical *Mutator* element was discovered in a maize stocks showing a high forward mutation rate (Robertson 1978). Since then, many *Mutator*-like elements (MULEs) have been identified in plants (Holligan et al. 2006), fungi (Chalvet et al. 2003), protozoans (Pritham et al. 2005; Lopes et al. 2009), and metazoans (Marquez and Pritham 2010). Autonomous *MuDR*-like elements carry two open reading frames, *mudrA* and *mudrB*, the former coding for a transposase, while a function of the latter is not well defined. There is also a group of *Mutator*-like autonomous elements, e.g. *Jittery*, carrying only *mudrA*-like ORF (Xu et al. 2004).

Tandemly repeated motifs of 2–6 nt are commonly referred to as microsatellites. Microsatellites exhibit variation in length, structure, frequency of individual motifs and genomic distribution (Schulman et al. 2005). In plants, (TA)_n_ repeats are more abundant compared to other dinucleotide motifs (Wang et al. 1994). Microsatellite regions are considered as hypervariable, as the number of tandem repeats can be changed following DNA polymerase slippage in the course of DNA replication. In plants, tandem repeats were shown to be preferentially associated with gene-rich regions (Morgante et al. 2002). In *Medicago truncatula*, microsatellites were found near genes, in 5′ and 3′ untranslated regions (UTRs) and introns (Mun et al. 2006).

Relationships between microsatellites and TEs were reported in insects (Meglécz et al. 2007), nematodes (Johnson et al. 2006), and plants (Temnykh et al. 2001; Tero et al. 2006). TEs targeting microsatellites were reported in *Lepidoptera* (Coates et al. 2010), rice (Akagi et al. 2001), and maize (Wang and Dooner 2006). Protomicrosatellites were shown to be created by *Angela* LTR retrotransposons in pea (Smýkal et al. 2009). Microsatellite-associated interspersed nuclear elements (MINEs) containing hitchhiking microsatellites, identified in moths (Coates et al. 2011) are another example of possible TE-microsatellite relationships.

Here, we report on *MuTAnTs*, a novel family of MULEs present in *M. truncatula* and targeting (TA)_n_ microsatellite repeats. We identified 218 copies of *MuTAnTs* and characterized a putative autonomous element carrying a complete ORF encoding a *mudrA*-like transposase.

## Materials and methods

### Plant material

Molecular analyses were performed on the reference line A17 ‘*Jemalong*’, 2HA (an A17 derivative) and on 21 wild accessions of *M. truncatula* provided by INRA, Montpelier, France. Apart from *M. truncatula*, eight other Fabaceae species, i.e. *Lupinusangustifolius* L., *L. luteus* L., *Pisum sativum* L., *Phaseolus vulgaris* L., *Trifolium pratense* L., *T. repens* L., and *Vicia faba* L. were included in the analyses (Additional file 1). Each accession was represented by a single plant, seeds were germinated according to the *Medicago* Handbook (Garcia et al. 2006), plants were grown in pots in the greenhouse. Genomic DNA was isolated from the fresh tissue collected from ca. 8-weeks-old plants with Plant DNeasy Mini Kit (Qiagen) following the manufacturer’s protocol.

### Mining for *MuTAnT* copies in *M. truncatula*

The family of non-autonomous elements flanked by TA repeats was identified following manual inspection of TE sequences reported by REPET (Flutre et al. 2011) for *M. truncatula* genome version 3.5.2 (Young et al. 2011) downloaded from medicago.org. Individual copies of *MuTAnTs* were mined with TARGET (Han et al. 2009) at www.iplantcollaborative.org using a *MuTAnT* sequence reported by REPET as a query. The related autonomous element was identified with TIRfinder (Gambin et al. 2013) using the following parameters: tirMask: GGGGTTTGCTAGAACA, tsdMask: N, tirSeqMismatches: 1, tsdSeqMismatches: 1, tirMaskMismatches: 3, tsdMaskMismatches: 0, and the aminoacid sequence of the maize *mudrA* transposase (Genebank acc. no. AAA21566) as a query with tblastn threshold of 1e-2. *MuTAnT* structure was analysed with mfold (Zuker 2003) and Dotlet (Junier and Pagni 2000). Sequence logos of TIRs were obtained with WebLogo (Crooks et al. 2004).

### *MuTAnT* diversity and evolutionary dynamics

Sequences were processed with BioEdit (Hall 1999). Phylogenetic analyses including calculation of pairwise distances under Tajima–Nei model were performed with MEGA 5.2 (Tamura et al. 2011), frequencies were calculated with MS Excel. Possibility of past transposition events was demonstrated through the identification of sequences related to empty sites (RESites), which are paralogous sequences lacking TE insertion. Basic strategy included the comparison between the occupied locus and related empty sequence reveals the TSD events and gaps corresponding to the TE insertion (Le et al. 2000).

### PCR assay

The PCR assay was used to investigate the distribution of *AutoMuTAnT* copies within *Fabaceae*, as well as to reveal the genomic distribution of *MuTAnTs* among *M*. *truncatula* ecotypes. Primers were designed using Primer3 (Koressaar and Remm 2007; Untergasser et al. 2012). PCR reactions were set up as followed: ca. 10 ng of genomic DNA, 0.5 mM dNTP, 0.4 µM of forward primer, 0.4 µM of reversed primer, 5 % of DMSO, 1× buffer for AccuTaq LA DNA Polymerase, 0.2 U of JumpStart AccuTaq LA DNA Polymerase Mix (Sigma Aldrich) in the total volume of 20 µl (Additional file 1). The following PCR conditions were applied: 96 °C/30 s, 30 × (94 °C/15 s, 62 °C/30 s, 68 °C/2 min), and 68 °C/30 min. Amplified fragments were separated in 1.5 % agarose gel in 1× TBE buffer and detected by ethidium bromide staining. Target bands were extracted from the gel with Wizard SV Gel and PCR Clean-Up System (Promega) as described by the manufacturer. Purified fragments were ligated into pGEM-T vector and cloned into *E. coli* strain *DH10B* according to standard cloning procedure provided by Promega. Positive clones verified by PCR assay were Sanger-sequenced in Genomed SA, Warsaw, Poland.

## Results

### Identification and characterization of *MuTAnTs*

**Fig. 1:**
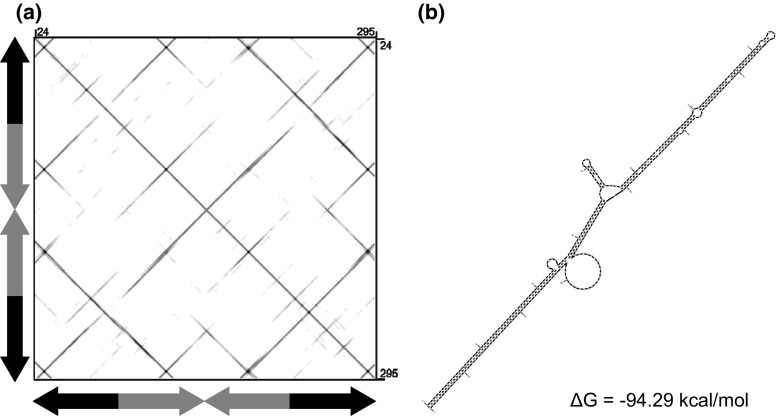
Structure of *MuTAnT* elements. **a** Self-alignment dot-plot illustrating the structure of *MuTAnT.* Arrows on *X* and *Y* axes depict orientation of the four similar segments. **b** The foldback structure predicted with mfold

**Fig. 2:**
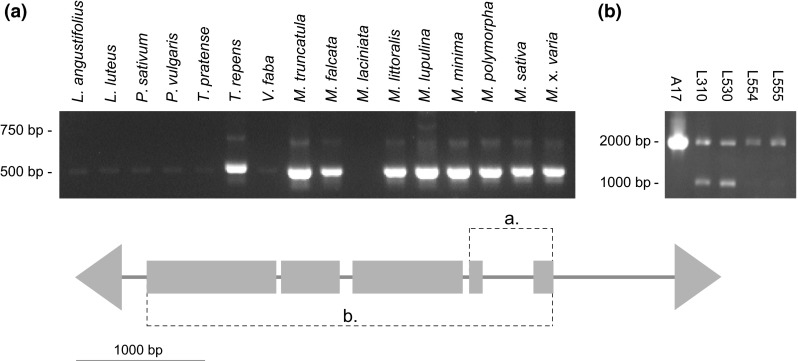
PCR assay within DDE/DDD domain of the *AutoMuTAnT* transposase. Results of PCR assay with primers anchored in two regions of the DDE/DDD domain within selected *Fabaceae* species (**a**) and *M. truncatula* ecotypes (**b**)

Upon visual inspection of REPET output for *M. truncatula*, we identified a 292 bp-long element flanked by TA repeats. It comprised almost identical 144 bp-long TIRs spawning a foldback sequence, which showed a strong propensity to form a hairpin-like tertiary structure (Fig. 1). We identified 218 related elements ranging from ca. 200 bp to over 1.6 kb and grouped them into a family named *MuTAnT* (*Mutator*-like (TA)_n_Targeting). A significant fraction (90 %) of these elements ranged in size from 200 to 300 bp, the mean TIR length was 47 bp (Additional file 2). A related 4873 bp-long putative autonomous element, dubbed *AutoMuTAnT* (position 26,442,402–26,447,275 on chromosome 1), carried a single ORF composed of five exons, predicted to encode a 833 aa protein (Fig. 2) similar to *Jittery* transposase (e-value: 6e-79, GenBank acc. no. AAF66982, Xu et al. 2004). The insertion was flanked by long TA stretches comprising 59 and 27 perfect repeats on the 5′ and 3′ end, respectively. The sequence of *AutoMuTAnT* and majority rule consensus sequences of *MuTAnT*1 and *MuTAnT*2 subfamilies are provided in Additional file 3.

*AutoMuTAnTs* were present only within the Trifolieae tribe of the Fabaceae family, including white clover and all *Medicago* spp. but *M. laciniata* (Fig. 2a). Two wild ecotypes, *L310* and *L530*, possibly carried other, likely truncated, copies of *AutoMuTAnT*, not present in the reference genome of A17 cv. ‘*Jemalong*’ (Fig. 2b).

### Diversity of *MuTAnTs*

**Fig. 3:**
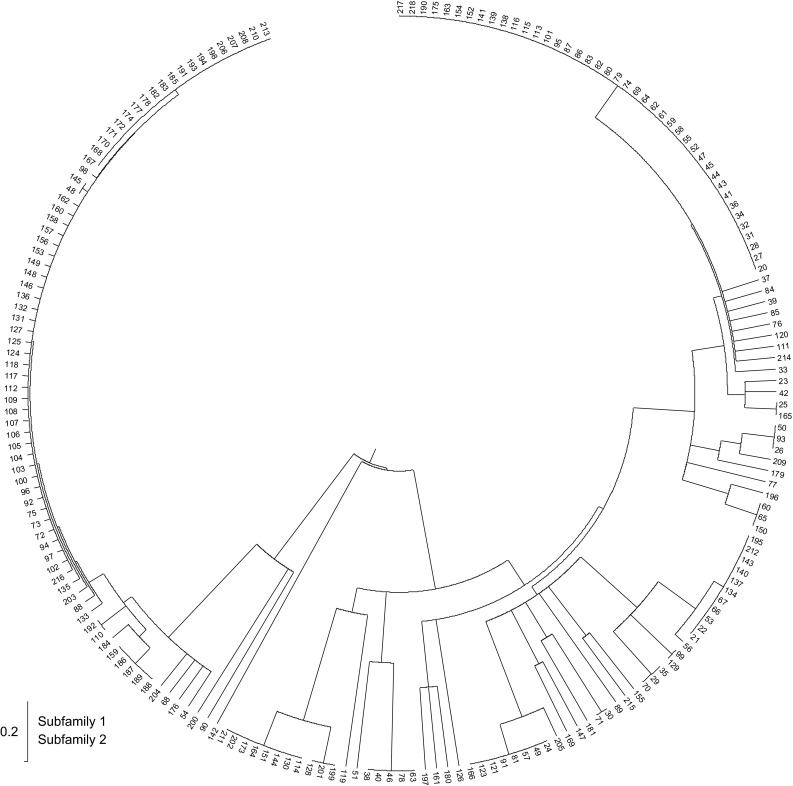
Neighbor-joining tree representing diversity of 148 *MuTAnTs*

**Fig. 4:**
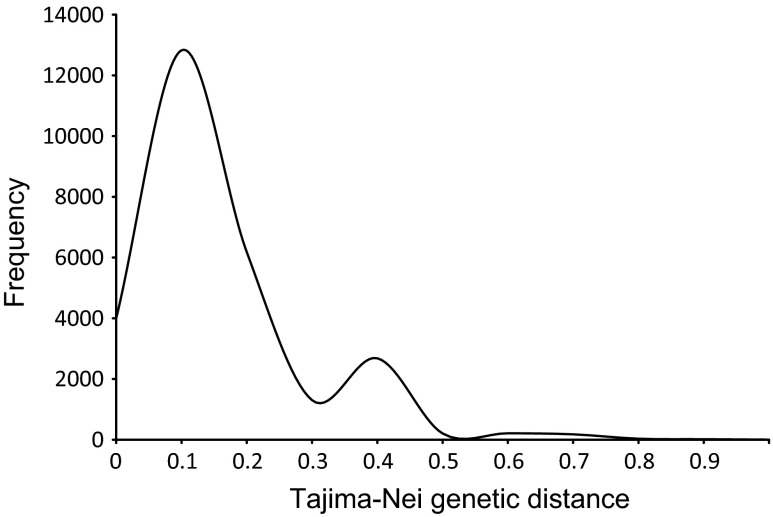
Within family frequency of Tajima–Nei pairwise distances between *MuTAnT* copies

Within the *MuTAnT* copies mined from the A17 genome assembly, twenty carried nested insertions or rearrangements. The remaining copies could be divided into two subfamilies, *MuTAnT*1 and *MuTAnT*2, grouping 74 and 124 elements, respectively (Fig. 3). The frequency of pairwise genetic distances calculated for all copies drawn as a histogram showed a bimodal distribution (Fig. 4). Fifty of the 198 elements were defective, completely or partially lacking one of TIRs (Additional file 4). Complete copies differed in terms of presence of the distal GGGG/CCCC stretches, as the number of G or C varied from zero to four.

**Fig. 5:**
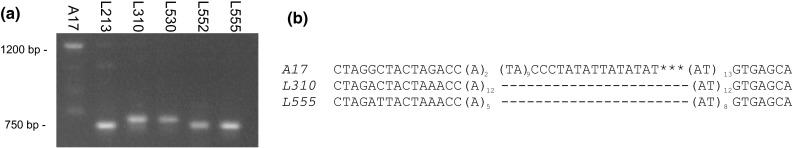
PCR assay of the *MuTAnT*-*32* insertion site. **a** Results of the PCR assay for five wild *M. truncatula* ecotypes and the reference line A17 with primers flanking the *MuTAnT*-32 insertion site. **b** Alignment of the corresponding empty and occupied variants at the *MuTAnT*-32 insertion site. The number of TA repeats is given in the subscript, the position of *MuTAnT*-32 is marked by *three asterisks*

In order to further analyze the distribution of *MuTAnT* insertions among *M. truncatula* accessions, we screened fourteen insertion sites using a PCR assay. In principle, length of the PCR product obtained with primers flanking the insertion site should indicate the presence or absence of a MITE copy. PCR amplification of regions comprising insertion sites was observed in 10 of 14 sites, all of them being polymorphic with respect to *MuTAnT* insertions (Fig. 5). In seven insertion sites, *MuTAnT* copies were present both in A17 and wild ecotypes, while in the remaining three sites, the insertions were unique for A17.

### Insertion site preference

**Fig. 6:**
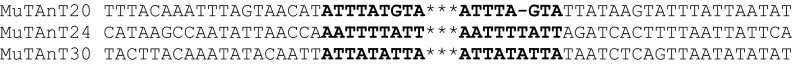
Target site duplication of four *MuTAnT* insertions. Target site duplications are in *bold font*, positions of *MuTAnT* insertions are marked by *three asterisks*

*MuTAnTs* were evenly distributed across chromosomes of *M. truncatula* with one insertion per 1.44 Mb on average. A substantial fraction of insertions were present in gene-rich regions, 36 of 218 identified copies occurred in introns or 5′ and 3′ UTRs, while additional 65 insertions were localized less than 1 kb away from genes. All 218 *MuTAnT* insertions occurred in AT-rich regions, predominantly inside (TA)_n_ microsatellites varying in length and reaching up to 41 repeats. An average microsatellite flanking a *MuTAnT* insertion in the A17 reference genome consisted of 11.7 (±9.5 SD) TA repeats. Only 14 of the 218 copies were not inserted into perfect (TA)_n_ microsatellites. For these, the surrounding sequences indicated presence 9 nt-long target site duplications (Fig. 6).

Additional insertion sites PCR-amplified from *M. truncatula* ecotypes carried on average 15 TA repeats on each TE flank. In contrast, sequencing of a subset of corresponding empty insertion sites and RESite analysis indicated that empty target sites consisted of nine TA repeats on average (Table 1).

**Table 1:** Number of TA repeats surrounding *MuTAnT* insertion sites on 5′ and 3′ insertion flanks and corresponding empty sites in *M. truncatula* ecotypes and A17 cv. ‘*Jemalong’*

Element	*M. truncatula* ecotypes	Number of TA repeats		
		Occupied site		Empty site
		5′	3′	
*MuTAnT*-*22*	A17	25	6	–
	L310	–	–	9
	530	–	–	7
	L555	8	111	–
*MuTAnT*-*25*	A17	10	10	–
	L310	–	–	12
	L555	–	–	8
*MuTAnT*-*33*	A17	21	9	–
	L310	–	–	9
	L530	–	–	4
*MuTAnT*-*42*	A17	11	5	–
	L554	13	2	–
*MuTAnT*-*47*	A17	11	12	–
	L213	8	12	–
	L555	9	12	–
*MuTAnT*-*52*	A17	6	22	–
	L310	6	13	–
	L554	3	12	–
	L555	7	9	–
*MuTAnT*-*63*	A17	24	22	–
	L555	–	–	11

## Discussion

We identified and characterized a novel family of MULEs named *MuTAnT* showing a strong preference for insertion into (TA)_n_ microsatellites. We found *MuTAnTs* to be composed of long TIRs built up from modules forming a foldback structure, characteristic to previously reported families, such as *Jittery* in maize flanked by 181 bp-long TIRs (Xu et al. 2004) or *FARE1* in *Arabidopsis*, a group of *Foldback* carrying long palindromic repeats on both ends (Windsor and Waddell 2000), currently classified as MULEs (Feschotte and Pritham 2007). The appurtenance of *MuTAnTs* to MULEs is further supported by similarity of the *mudrA*-like protein sequence of *AutoMuTAnT* and the *Jittery* transposase. The presence of *AutoMuTAnT* in white clover and in all but one analyzed *Medicago* species demonstrates that *MuTAnTs* are likely to predate the origin of the *Medicago* genus, as they were present in the most recent common ancestor of *Trifolium* and *Medicago* which lived at least 16 million years ago (Lavin et al. 2005).

To reveal the evolutionary history of the *MuTAnT* family, we calculated frequencies of pairwise distances between elements, as proposed previously for DINE-1 elements in *Drosophila* (Yang et al. 2006) and *ATons* in yellow fever mosquito (Yang et al. 2012). It indicated two bursts of transpositional activity giving rise to two subfamilies. We also analyzed the distribution of *MuTAnT* copies among wild ecotypes of *M*. *tuncatula* by a PCR assay. Insertion polymorphisms of ten sites and the unique presence of *MuTAnT* copies in three of those sites in A17 are indicative for recent transposition events similar to those reported previously for other plant species and TE families (Naito et al. 2006; Benjak et al. 2009; Grzebelus et al. 2009, 2011).

Precise determination of TSDs for most copies was impeded due to the repetitive nature of the flanking sequences. In addition, a high proportion of defective copies was revealed, as 25 % of all identified *MuTAnTs* lacked a significant portion of one or both TIRs. Even if both TIRs were present, they varied in terms of deletions of one or more of the four distal nucleotides of TIRs (GGGG/CCCC). Nevertheless, we showed that *MuTAnTs* generated 9 nt-long TSD, which in general is typical for MULEs.

*MuTAnT* insertion sites are frequently located in proximity to genes, mostly less than 3 kb downstream or upstream from the adjacent coding region, with the number of insertions decreasing with the distance from genes. A similar tendency for insertion into gene-rich regions was reported early with the discovery of MITEs (Bureau and Wessler 1992, 1994) and was supported by subsequent studies (Yang et al. 2001; Sampath et al. 2013). As *MuTAnTs* are short, non-autonomous and relatively numerous in *M. truncatula*, they resemble MITEs both in terms of their structure and mode of operation. Notably, all *MuTAnT* insertions present within transcribed regions were located in UTRs or introns.

A hallmark of *MuTAnTs* activity is their propensity to insert into (TA)_n_ microsatellites. Affinity to insert into (TA)_n_ microsatellites provides a direct barrier against their insertions into coding regions. the apparent preference of *MuTAnTs* for insertion into TA repeats is possibly their survival strategy. Weak selection pressure imposed on microsatellite sites may favor TE families adapted to target microsatellites and use them as ‘safe havens.’ On the other hand, insertions proximal to coding regions can still introduce more subtle regulatory changes on the expression of adjacent genes. It is interesting to compare *MuTAnTs* to *AhMITEs,* a family of short MULEs inserting into AT-rich but non-microsatellite regions of the peanut genome (Shirasawa et al. 2012). Notably, we observed a similar behavior also in a minor group of *MuTAnTs*. Thus, it is possible that both families represent successive stages of the evolutionary process of exploiting microsatellites as target sites, with *AhMITEs* being a transitional form. *TAFT* and *Micron* elements identified in maize and rice, respectively, also show preference for insertion into (TA)_n_ microsatellites which suggests that the strategy may be more widespread, as it evolved independently in several unrelated families of DNA transposons.

## Electronic supplementary material

Supplementary File 1. List of plant materials and primers used in the study. (PDF 286 kb)

Supplementary File 2. Complete list of physical positions of *MuTAnTs* in *M. truncatula* A17. The complete information on physical positions with reference to distances from coding regions, number of TA repeats surrounding insertions and length of TIRs. (XLSX 25 kb)

Supplementary File 3. Sequence of *AutoMuTAnT* and consensus sequences of *MuTAnT*1 and *MuTAnT*2. (FAS 5 kb)

Supplementary File 4. Grahpical visualizations. Sequence logos of TIRs obtained with WebLogo and graphical visualization of *MuTAnT* family members reported by TARGeT. (PDF 3193 kb)
