# Supplementary material for: MuTAnT: a family of Mutator-like transposable elements targeting TA microsatellites in Medicago truncatula
Source: Genetica. 2015 May 17;143(4):433–40. doi: 10.1007/s10709-015-9842-5 (PMC4486113; doi:10.1007/s10709-015-9842-5)
Supplement: Supplementary file 1 — List of plant materials and primers used in the study. (PDF 286 kb) [file 10709_2015_9842_MOESM1_ESM.pdf]

Supplementary table 1. Complete list of plant material used in the study.

**Genus**	**Section**	**Subsection**	**Species**	**Accession code**
Medicago	*Spirocarpos*	*Leptospireae*	*M. minima* (L.) Bartal. (eng. little medic)	243205[1]
Medicago	*Spirocarpos*	*Leptospireae*	*M. laciniata* (L.) Mill. (eng. cut-leaf medic)	243370[1]
Medicago	*Spirocarpos*	*Leptospireae*	*M. polymorpha* L. (eng. bur medic)	243207[1]
Medicago	*Spirocarpos*	*Pachyspireae*	*M. littoralis* Rohde ex Loisel. (eng. strand medic)	243371[1]
Medicago	*Spirocarpos*	*Pachyspireae*	*M. truncatula* Gaertn. (eng. barrel medic)	A17 "*Jemalong*"[2]
Medicago	*Spirocarpos*	*Pachyspireae*	*M. truncatula* Gaertn. (eng. barrel medic)	L049[2]
Medicago	*Spirocarpos*	*Pachyspireae*	*M. truncatula* Gaertn. (eng. barrel medic)	L144[2]
Medicago	*Spirocarpos*	*Pachyspireae*	*M. truncatula* Gaertn. (eng. barrel medic)	L163[2]
Medicago	*Spirocarpos*	*Pachyspireae*	*M. truncatula* Gaertn. (eng. barrel medic)	L174[2]
Medicago	*Spirocarpos*	*Pachyspireae*	*M. truncatula* Gaertn. (eng. barrel medic)	L213[2]
Medicago	*Spirocarpos*	*Pachyspireae*	*M. truncatula* Gaertn. (eng. barrel medic)	L245[2]
Medicago	*Spirocarpos*	*Pachyspireae*	*M. truncatula* Gaertn. (eng. barrel medic)	L290[2]
Medicago	*Spirocarpos*	*Pachyspireae*	*M. truncatula* Gaertn. (eng. barrel medic)	L310[2]
Medicago	*Spirocarpos*	*Pachyspireae*	*M. truncatula* Gaertn. (eng. barrel medic)	L321[2]
Medicago	*Spirocarpos*	*Pachyspireae*	*M. truncatula* Gaertn. (eng. barrel medic)	L337[2]
Medicago	*Spirocarpos*	*Pachyspireae*	*M. truncatula* Gaertn. (eng. barrel medic)	L369[2]
Medicago	*Spirocarpos*	*Pachyspireae*	*M. truncatula* Gaertn. (eng. barrel medic)	L530[2]
Medicago	*Spirocarpos*	*Pachyspireae*	*M. truncatula* Gaertn. (eng. barrel medic)	L543[2]
Medicago	*Spirocarpos*	*Pachyspireae*	*M. truncatula* Gaertn. (eng. barrel medic)	L544[2]
Medicago	*Spirocarpos*	*Pachyspireae*	*M. truncatula* Gaertn. (eng. barrel medic)	L552[2]
Medicago	*Spirocarpos*	*Pachyspireae*	*M. truncatula* Gaertn. (eng. barrel medic)	L554[2]
Medicago	*Spirocarpos*	*Pachyspireae*	*M. truncatula* Gaertn. (eng. barrel medic)	L555[2]
Medicago	*Spirocarpos*	*Pachyspireae*	*M. truncatula* Gaertn. (eng. barrel medic)	L557[2]
Medicago	*Spirocarpos*	*Pachyspireae*	*M. truncatula* Gaertn. (eng. barrel medic)	L648[2]
Medicago	*Spirocarpos*	*Pachyspireae*	*M. truncatula* Gaertn. (eng. barrel medic)	A20[2]
Medicago	*Spirocarpos*	*Pachyspireae*	*M. truncatula* Gaertn. (eng. barrel medic)	2HA[2]
Medicago	*Spirocarpos*	*Pachyspireae*	*M. truncatula* Gaertn. (eng. barrel medic)	R108[2]
Medicago	*Lupularia*	–	*M. lupulina* L. (eng. black medic)	243372[1]
Medicago	*Medicago*	–	*M. sativa* L. (eng. alfalfa)	243208[1]
Medicago	*Medicago*	–	*M.* x *varia* Martyn. (eng. bastard medic)	243096[1]
Medicago	*Medicago*	–	*M. falcata* L. (eng. sickle alfalfa)	243267[1]
Lupinus	–	–	*L. angustifolius* L. (eng. narrow-leaf lupin)	Kardyl[3]
Lupinus	–	–	*L. luteus* L. (eng. yellow lupin)	Dukat[3]
Pisum	–	–	*P. sativum* L. (eng. pea)	Cysterski[3]
Phaseolus	–	–	*P. vulgaris* L. (eng. bean)	Złota saxa[3]
Trifolium	*Trifolium*	–	*T. pratense* L. (eng. cowgrass clover)	243118[1]
Trifolium	*Trifoliastrum*	–	*T. repens* L. (eng. Dutch clover)	243125[1]
Vicia	*Faba*	–	*V. faba* L. (eng, bell-bean)	Bizon[3]

[1] National Center for Plant Genetic Resources at the The Plant Breeding and Acclimatization Institute, Radzikow, Poland
[2] French National Institute for Agricultural Research, Montpelier, France
[3] PlantiCo, Zielonki, Poland

Supplementary Table 2. Complete list of primers used in the study.

Insertion sites	Primers (5'-3')	
**22**	CCCTAGGTTTGCCATGTTGT	CGACTTGGTGGCCACTTTAT
**23**	GGCGTTGAACCTTCATGATT	TCTGCCGGTCCCATAACTTA
**25**	CTTGCTTTTGTTGTGGGGTA	TCAAAGAATTCTAACCGCCG
**32**	CCGACGACATCGTGTCCCGTCTTTCCAA	CATTCCAAGGCCACCACCAACGTC
**33**	AACTAATTTCCCCTGATAACTGTAGA	AAACCATAACGTAAGTGAAAAACTAAC
**36**	CGAATTAACTCCGATGCTGA	ACACTGCCATAAGGCTCCAC
**41**	TTGCTCCATTTGTTCTCACG	GTGGAAGGCATGGATTTTGT
**42**	AGCGGTGCTAAATCAGAGGA	AGATTTTTGACCCGTGCTCC
**47**	AGTTGTTGGTTCTTGGATCCATTT	CAAGAAACTCTTCTTACAATTTTGTTT
**52**	TAGAAATACAATGACATGTGGGATG	AGACATAGAGATTGGAAATTGTTGC
**63**	TTGACAATGAGAGGTTGACTTAAAA	TTTCTTTTTAAGAGGAATCAGTGACA
**76**	TCATTTTACCGGATTCTTTTTCA	GAACCCATTCAACAACACGA
**83**	CAACCTCGAACCTGACCAAT	GAAAAGGTTCCCAACGTGAA
**111**	CCCAGGAAATCTGGGAATTT	GGAAAAACATTGCCTTGGTG
**111**	GCTAACCAACTTCACTTCAAACAGT	ACGGCATAAAAGAGGAGAATAAACT
**Amplification of the DDE/DDD domain**	AATCGATGAAAATAACAAATTGGAG<br>TAAAATCATCGTATCTCGACCCTAA<br>AATCGATGAAAATAACAAATTGGAG	
