# Supplementary material for: MuTAnT: a family of Mutator-like transposable elements targeting TA microsatellites in Medicago truncatula
Source: Genetica. 2015 May 17;143(4):433–40. doi: 10.1007/s10709-015-9842-5 (PMC4486113; doi:10.1007/s10709-015-9842-5)
Supplement: Supplementary file 4 — Grahpical visualizations. Sequence logos of TIRs obtained with WebLogo and graphical visualization of MuTAnT family members reported by TARGeT. (PDF 3193 kb) [file 10709_2015_9842_MOESM4_ESM.pdf]

Supplementary figure 1. Sequence logos calculated for 50 bp long terminal parts of 198 *MuTAnTs* copies.

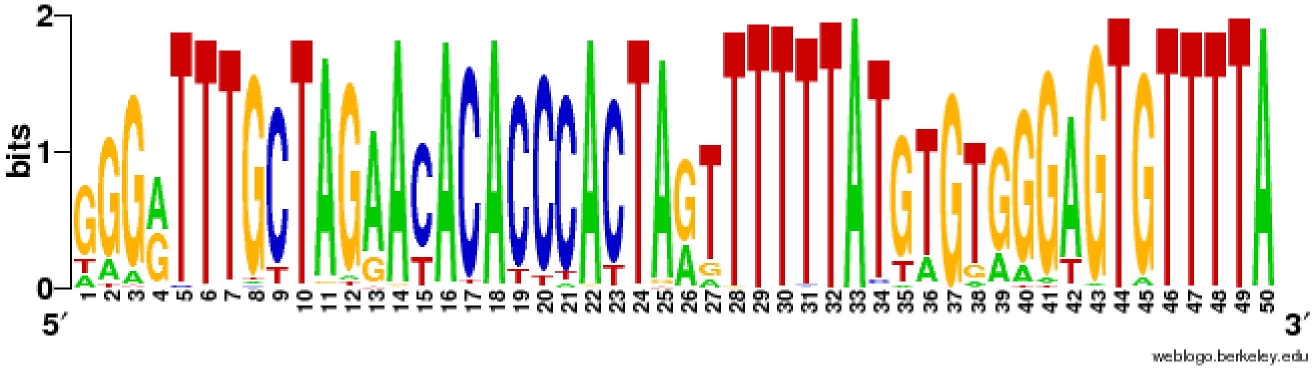

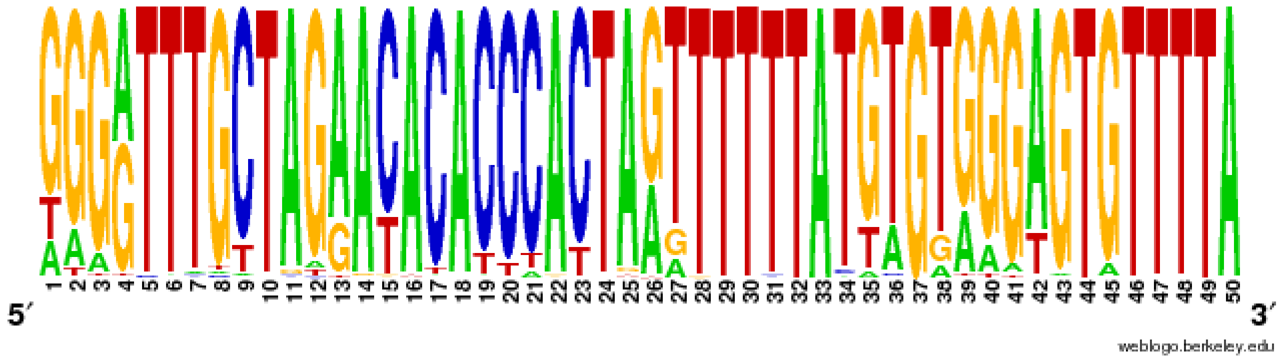

Supplementary figure 2. Graphical visualization of the blastn results of *MuTAnTs* family members obtained with TARGeT pipeline.

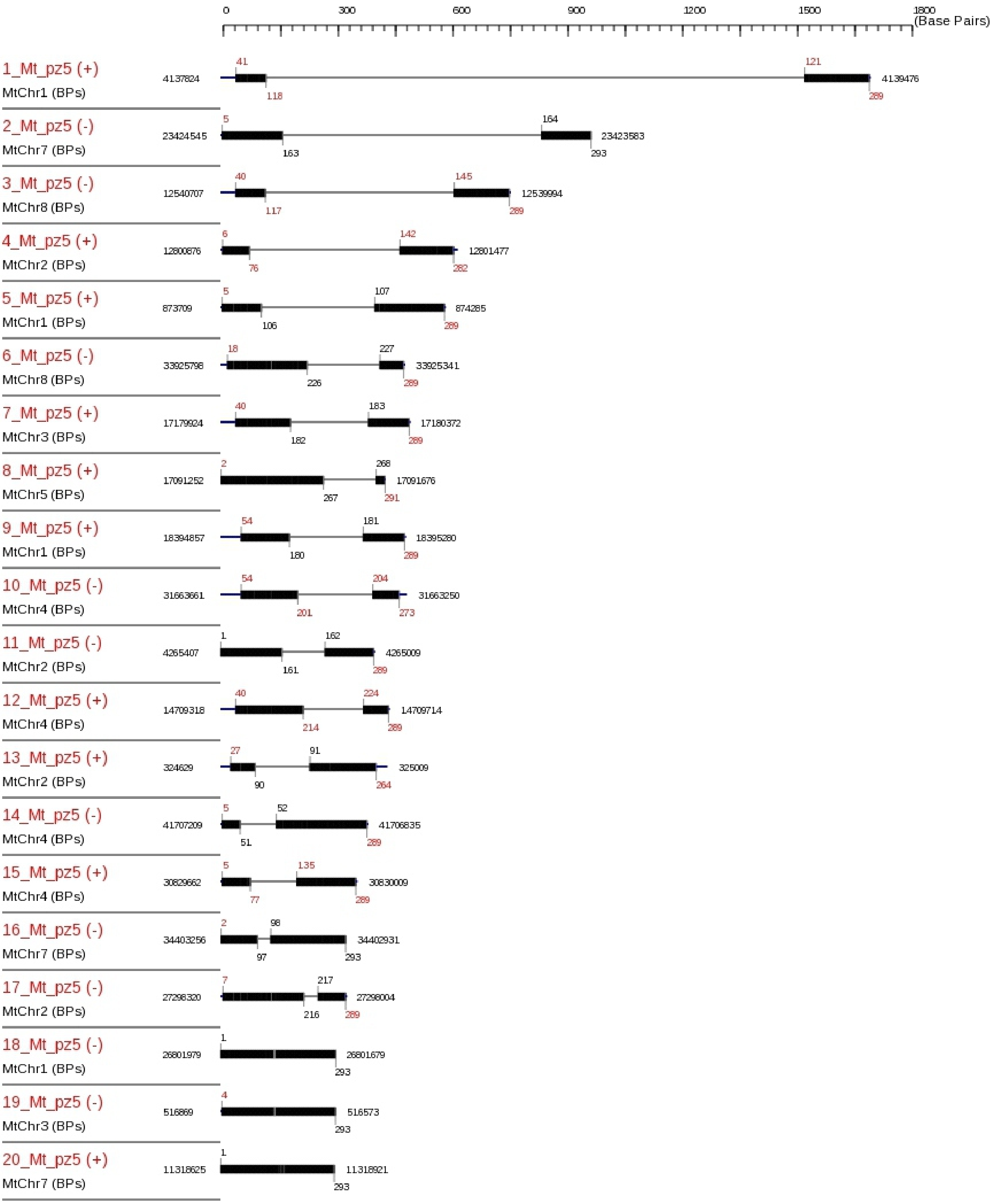

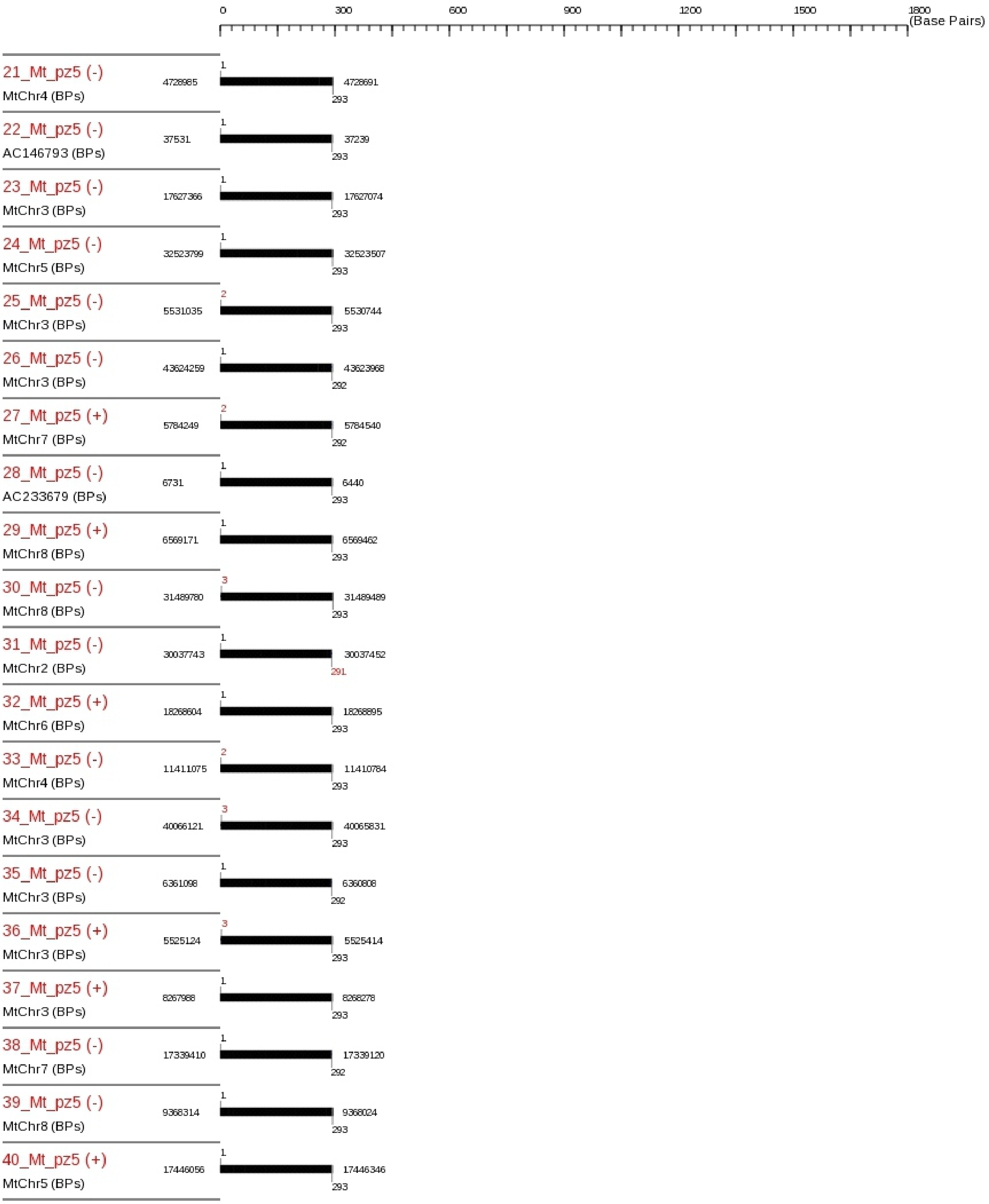
0
300
600
900
1200
1500
1800
(Base Pairs)
21_Mt_pz5 (-)
MtChr4 (BPs)
4728985 1 4728691 293
22_Mt_pz5 (-)
AC146793 (BPs)
37531 1 37239 293
23_Mt_pz5 (-)
MtChr3 (BPs)
17627366 1 17627074 293
24_Mt_pz5 (-)
MtChr5 (BPs)
32523799 1 32523507 293
25_Mt_pz5 (-)
MtChr3 (BPs)
5531035 2 5530744 293
26_Mt_pz5 (-)
MtChr3 (BPs)
43624259 1 43623968 292
27_Mt_pz5 (+)
MtChr7 (BPs)
5784249 2 5784540 292
28_Mt_pz5 (-)
AC233679 (BPs)
6731 1 6440 293
29_Mt_pz5 (+)
MtChr8 (BPs)
6569171 1 6569462 293
30_Mt_pz5 (-)
MtChr8 (BPs)
31489780 3 31489489 293
31_Mt_pz5 (-)
MtChr2 (BPs)
30037743 1 30037452 291
32_Mt_pz5 (+)
MtChr6 (BPs)
18268604 1 18268895 293
33_Mt_pz5 (-)
MtChr4 (BPs)
11411075 2 11410784 293
34_Mt_pz5 (-)
MtChr3 (BPs)
40066121 3 40065831 293
35_Mt_pz5 (-)
MtChr3 (BPs)
6361098 1 6360808 292
36_Mt_pz5 (+)
MtChr3 (BPs)
5525124 3 5525414 293
37_Mt_pz5 (+)
MtChr3 (BPs)
8267988 1 8268278 293
38_Mt_pz5 (-)
MtChr7 (BPs)
17339410 1 17339120 292
39_Mt_pz5 (-)
MtChr8 (BPs)
9368314 1 9368024 293
40_Mt_pz5 (+)
MtChr5 (BPs)
17446056 1 17446346 293

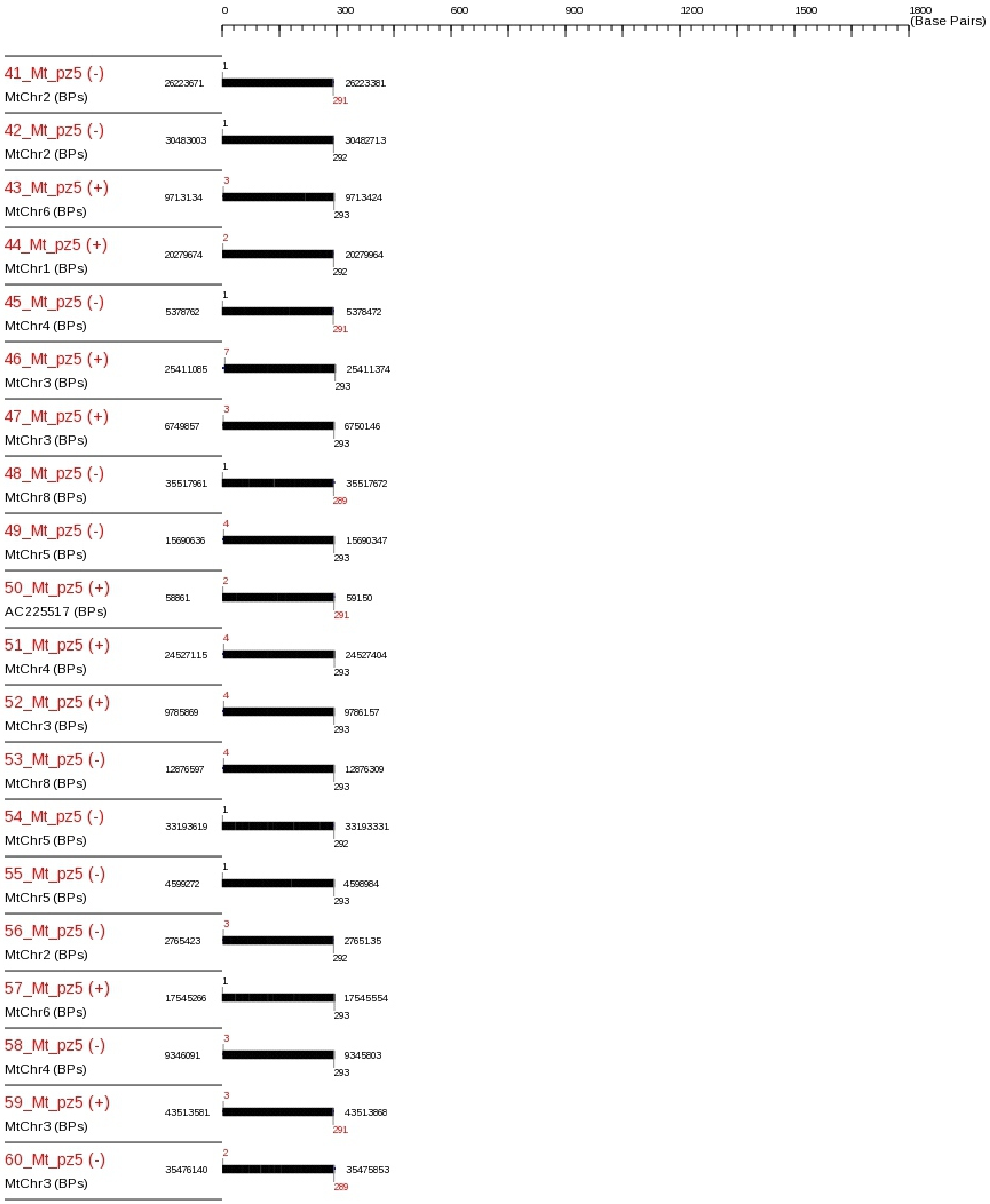
0
300
600
900
1200
1500
1800
(Base Pairs)
41_Mt_pz5 (-)
MtChr2 (BPs)
26223671
1
26223381
291
42_Mt_pz5 (-)
MtChr2 (BPs)
30483003
1
30482713
292
43_Mt_pz5 (+)
MtChr6 (BPs)
9713134
3
9713424
293
44_Mt_pz5 (+)
MtChr1 (BPs)
20279674
2
20279964
292
45_Mt_pz5 (-)
MtChr4 (BPs)
5378762
1
5378472
291
46_Mt_pz5 (+)
MtChr3 (BPs)
25411085
7
25411374
293
47_Mt_pz5 (+)
MtChr3 (BPs)
6749857
3
6750146
293
48_Mt_pz5 (-)
MtChr8 (BPs)
35517961
1
35517672
289
49_Mt_pz5 (-)
MtChr5 (BPs)
15690636
4
15690347
293
50_Mt_pz5 (+)
AC225517 (BPs)
58861
2
59150
291
51_Mt_pz5 (+)
MtChr4 (BPs)
24527115
4
24527404
293
52_Mt_pz5 (+)
MtChr3 (BPs)
9785869
4
9786157
293
53_Mt_pz5 (-)
MtChr8 (BPs)
12876597
4
12876309
293
54_Mt_pz5 (-)
MtChr5 (BPs)
33193619
1
33193331
292
55_Mt_pz5 (-)
MtChr5 (BPs)
4599272
1
4598984
293
56_Mt_pz5 (-)
MtChr2 (BPs)
2765423
3
2765135
292
57_Mt_pz5 (+)
MtChr6 (BPs)
17545266
1
17545554
293
58_Mt_pz5 (-)
MtChr4 (BPs)
9346091
3
9345803
293
59_Mt_pz5 (+)
MtChr3 (BPs)
43513581
3
43513868
291
60_Mt_pz5 (-)
MtChr3 (BPs)
35476140
2
35475853
289

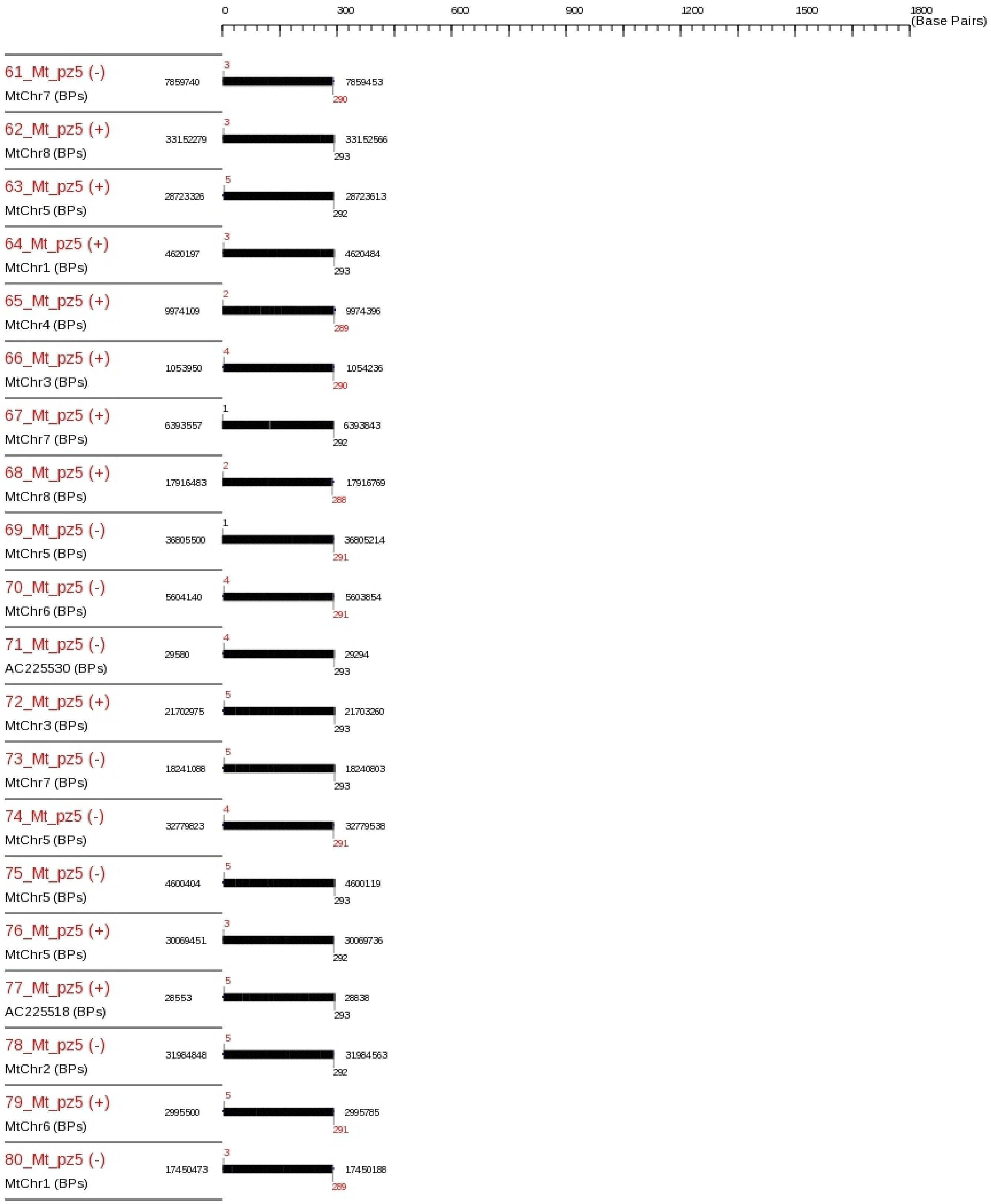
0
300
600
900
1200
1500
1800
(Base Pairs)
61_Mt_pz5 (-)
MtChr7 (BPs)
7859740
7859453
3
290
62_Mt_pz5 (+)
MtChr8 (BPs)
33152279
33152566
3
293
63_Mt_pz5 (+)
MtChr5 (BPs)
28723326
28723613
5
292
64_Mt_pz5 (+)
MtChr1 (BPs)
4620197
4620484
3
293
65_Mt_pz5 (+)
MtChr4 (BPs)
9974109
9974396
2
289
66_Mt_pz5 (+)
MtChr3 (BPs)
1053950
1054236
4
290
67_Mt_pz5 (+)
MtChr7 (BPs)
6393557
6393843
1
292
68_Mt_pz5 (+)
MtChr8 (BPs)
17916483
17916769
2
288
69_Mt_pz5 (-)
MtChr5 (BPs)
36805500
36805214
1
291
70_Mt_pz5 (-)
MtChr6 (BPs)
5604140
5603854
4
291
71_Mt_pz5 (-)
AC225530 (BPs)
29580
29294
4
293
72_Mt_pz5 (+)
MtChr3 (BPs)
21702975
21703260
5
293
73_Mt_pz5 (-)
MtChr7 (BPs)
18241088
18240803
5
293
74_Mt_pz5 (-)
MtChr5 (BPs)
32779823
32779538
4
291
75_Mt_pz5 (-)
MtChr5 (BPs)
4600404
4600119
5
293
76_Mt_pz5 (+)
MtChr5 (BPs)
30069451
30069736
3
292
77_Mt_pz5 (+)
AC225518 (BPs)
28553
28838
5
293
78_Mt_pz5 (-)
MtChr2 (BPs)
31984848
31984563
5
292
79_Mt_pz5 (+)
MtChr6 (BPs)
2995500
2995785
5
291
80_Mt_pz5 (-)
MtChr1 (BPs)
17450473
17450188
3
289

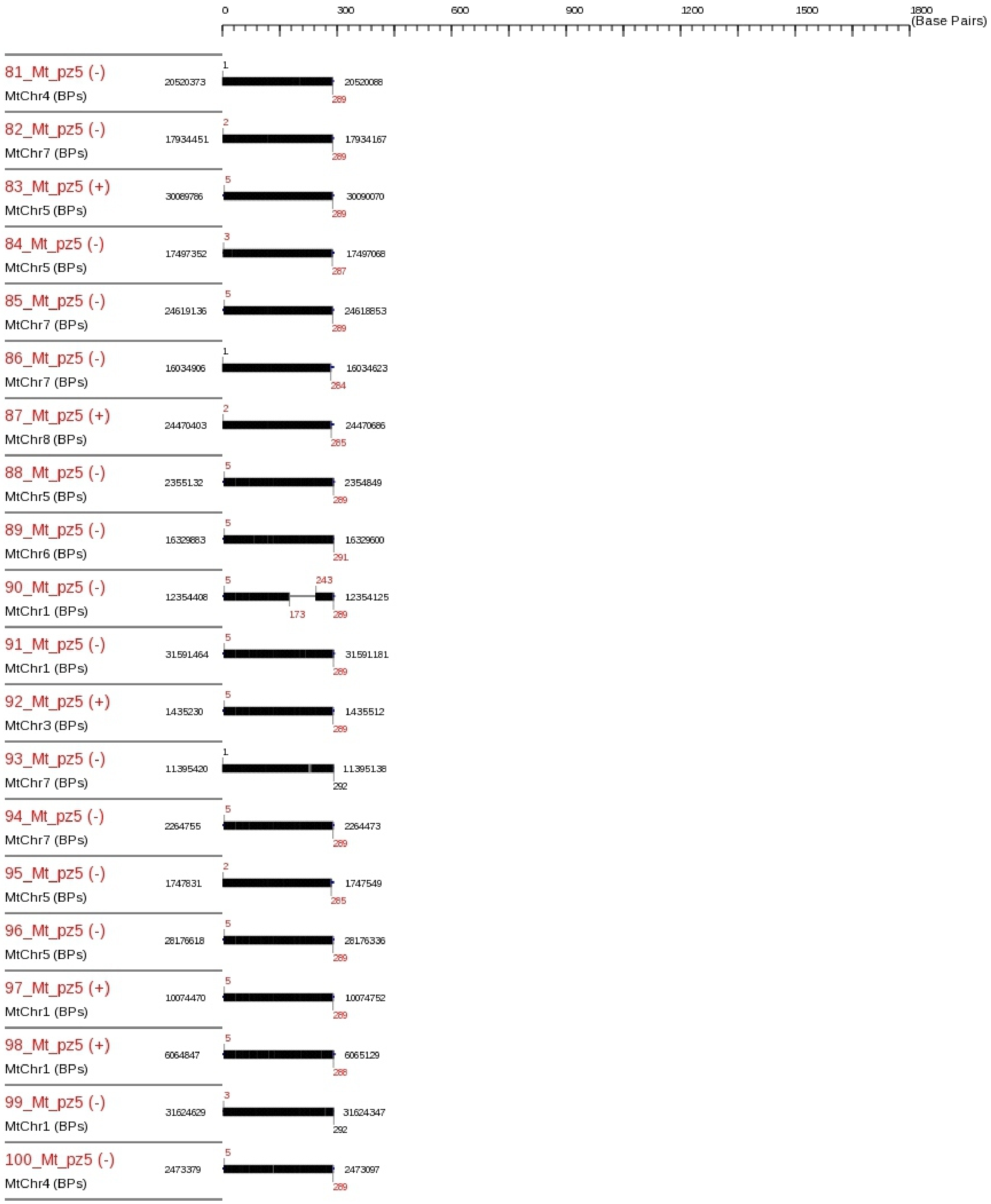
0
300
600
900
1200
1500
1800
(Base Pairs)
81_Mt_pz5 (-)
MtChr4 (BPs)
20520373
20520088
82_Mt_pz5 (-)
MtChr7 (BPs)
17934451
17934167
83_Mt_pz5 (+)
MtChr5 (BPs)
30089786
30090070
84_Mt_pz5 (-)
MtChr5 (BPs)
17497352
17497068
85_Mt_pz5 (-)
MtChr7 (BPs)
24619136
24618853
86_Mt_pz5 (-)
MtChr7 (BPs)
16034906
16034623
87_Mt_pz5 (+)
MtChr8 (BPs)
24470403
24470686
88_Mt_pz5 (-)
MtChr5 (BPs)
2355132
2354849
89_Mt_pz5 (-)
MtChr6 (BPs)
16329883
16329600
90_Mt_pz5 (-)
MtChr1 (BPs)
12354408
12354125
91_Mt_pz5 (-)
MtChr1 (BPs)
31591464
31591181
92_Mt_pz5 (+)
MtChr3 (BPs)
1435230
1435512
93_Mt_pz5 (-)
MtChr7 (BPs)
11395420
11395138
94_Mt_pz5 (-)
MtChr7 (BPs)
2264755
2264473
95_Mt_pz5 (-)
MtChr5 (BPs)
1747831
1747549
96_Mt_pz5 (-)
MtChr5 (BPs)
28176618
28176336
97_Mt_pz5 (+)
MtChr1 (BPs)
10074470
10074752
98_Mt_pz5 (+)
MtChr1 (BPs)
6064847
6065129
99_Mt_pz5 (-)
MtChr1 (BPs)
31624629
31624347
100_Mt_pz5 (-)
MtChr4 (BPs)
2473379
2473097

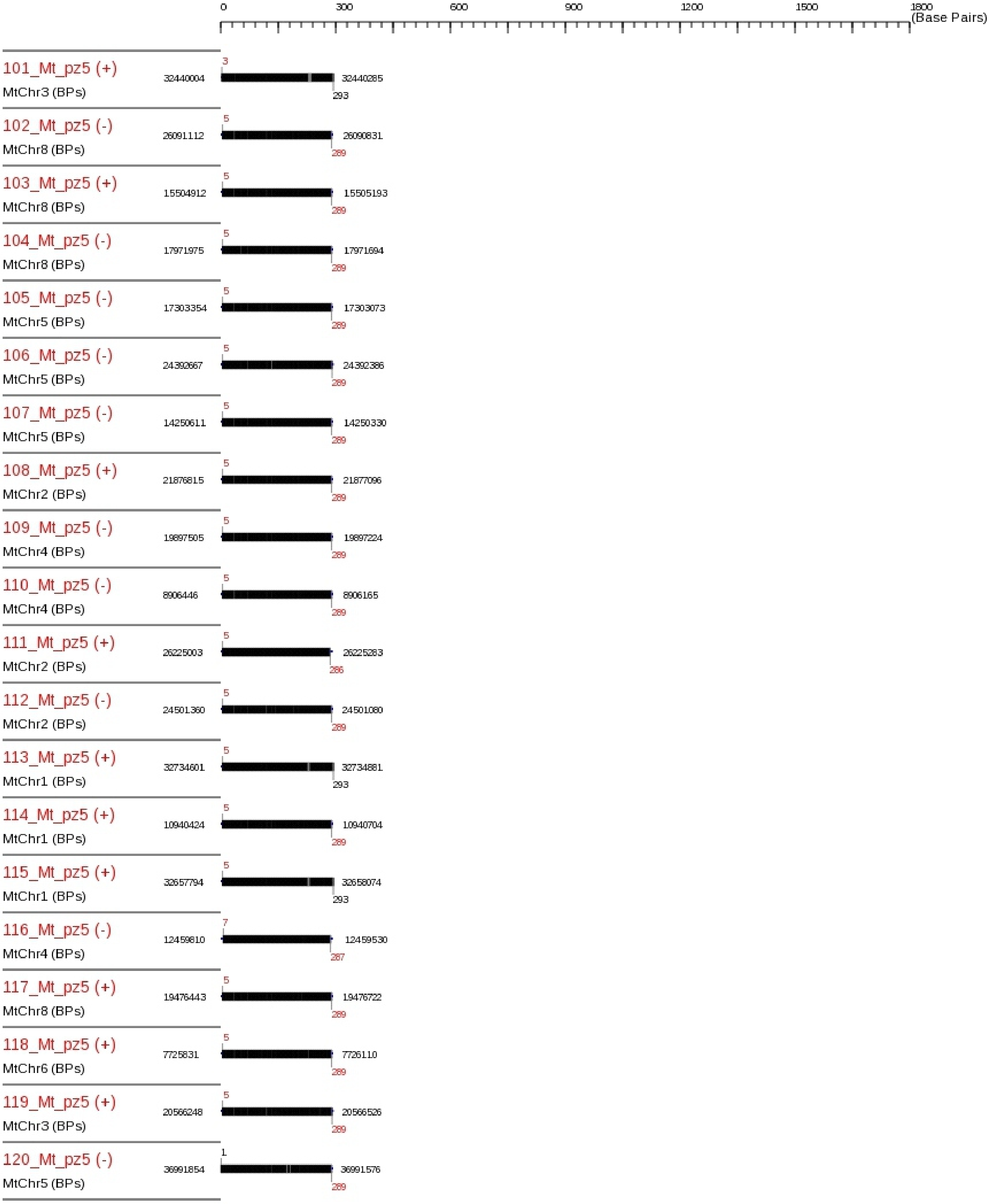
0
300
600
900
1200
1500
1800
(Base Pairs)
101_Mt_pz5 (+)
MtChr3 (BPs)
32440004
3
32440285
293
102_Mt_pz5 (-)
MtChr8 (BPs)
26091112
5
26090831
289
103_Mt_pz5 (+)
MtChr8 (BPs)
15504912
5
15505193
289
104_Mt_pz5 (-)
MtChr8 (BPs)
17971975
5
17971694
289
105_Mt_pz5 (-)
MtChr5 (BPs)
17303354
5
17303073
289
106_Mt_pz5 (-)
MtChr5 (BPs)
24392667
5
24392386
289
107_Mt_pz5 (-)
MtChr5 (BPs)
14250611
5
14250330
289
108_Mt_pz5 (+)
MtChr2 (BPs)
21876815
5
21877096
289
109_Mt_pz5 (-)
MtChr4 (BPs)
19897505
5
19897224
289
110_Mt_pz5 (-)
MtChr4 (BPs)
8906446
5
8906165
289
111_Mt_pz5 (+)
MtChr2 (BPs)
26225003
5
26225283
286
112_Mt_pz5 (-)
MtChr2 (BPs)
24501360
5
24501080
289
113_Mt_pz5 (+)
MtChr1 (BPs)
32734601
5
32734881
293
114_Mt_pz5 (+)
MtChr1 (BPs)
10940424
5
10940704
289
115_Mt_pz5 (+)
MtChr1 (BPs)
32657794
5
32658074
293
116_Mt_pz5 (-)
MtChr4 (BPs)
12459810
7
12459530
287
117_Mt_pz5 (+)
MtChr8 (BPs)
19476443
5
19476722
289
118_Mt_pz5 (+)
MtChr6 (BPs)
7725831
5
7726110
289
119_Mt_pz5 (+)
MtChr3 (BPs)
20566248
5
20566526
289
120_Mt_pz5 (-)
MtChr5 (BPs)
36991854
1
36991576
289

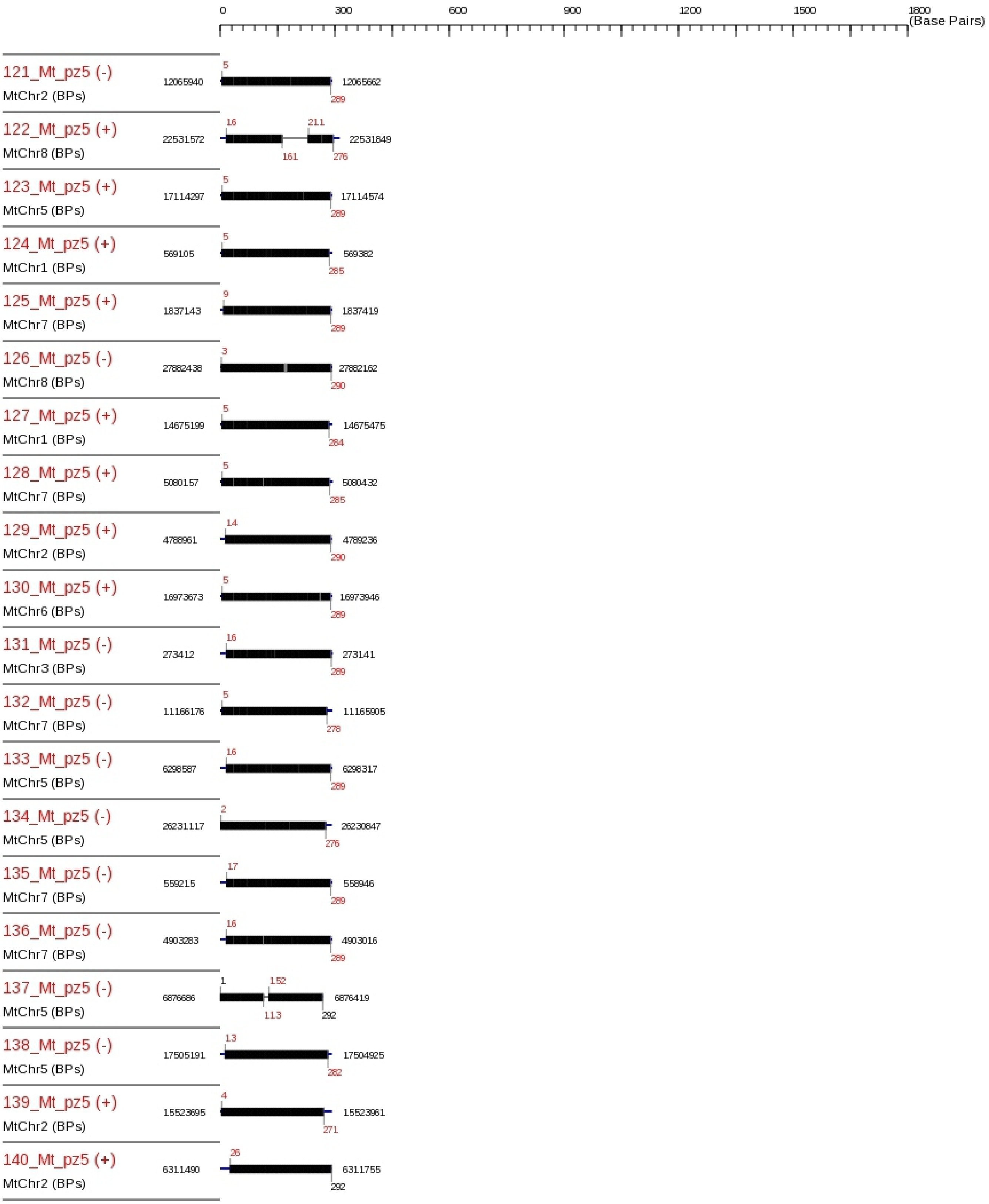
0
300
600
900
1200
1500
1800
(Base Pairs)
121_Mt_pz5 (-)
MtChr2 (BPs)
12065940
12065662
5
289
122_Mt_pz5 (+)
MtChr8 (BPs)
22531572
22531849
16
211
161
276
123_Mt_pz5 (+)
MtChr5 (BPs)
17114297
17114574
5
289
124_Mt_pz5 (+)
MtChr1 (BPs)
569105
569382
5
285
125_Mt_pz5 (+)
MtChr7 (BPs)
1837143
1837419
9
289
126_Mt_pz5 (-)
MtChr8 (BPs)
27882438
27882162
3
290
127_Mt_pz5 (+)
MtChr1 (BPs)
14675199
14675475
5
284
128_Mt_pz5 (+)
MtChr7 (BPs)
5080157
5080432
5
285
129_Mt_pz5 (+)
MtChr2 (BPs)
4788961
4789236
14
290
130_Mt_pz5 (+)
MtChr6 (BPs)
16973673
16973946
5
289
131_Mt_pz5 (-)
MtChr3 (BPs)
273412
273141
16
289
132_Mt_pz5 (-)
MtChr7 (BPs)
11166176
11165905
5
278
133_Mt_pz5 (-)
MtChr5 (BPs)
6298587
6298317
16
289
134_Mt_pz5 (-)
MtChr5 (BPs)
26231117
26230847
2
276
135_Mt_pz5 (-)
MtChr7 (BPs)
559215
558946
17
289
136_Mt_pz5 (-)
MtChr7 (BPs)
4903283
4903016
16
289
137_Mt_pz5 (-)
MtChr5 (BPs)
6876686
6876419
1
152
113
292
138_Mt_pz5 (-)
MtChr5 (BPs)
17505191
17504925
13
282
139_Mt_pz5 (+)
MtChr2 (BPs)
15523695
15523961
4
271
140_Mt_pz5 (+)
MtChr2 (BPs)
6311490
6311755
26
292

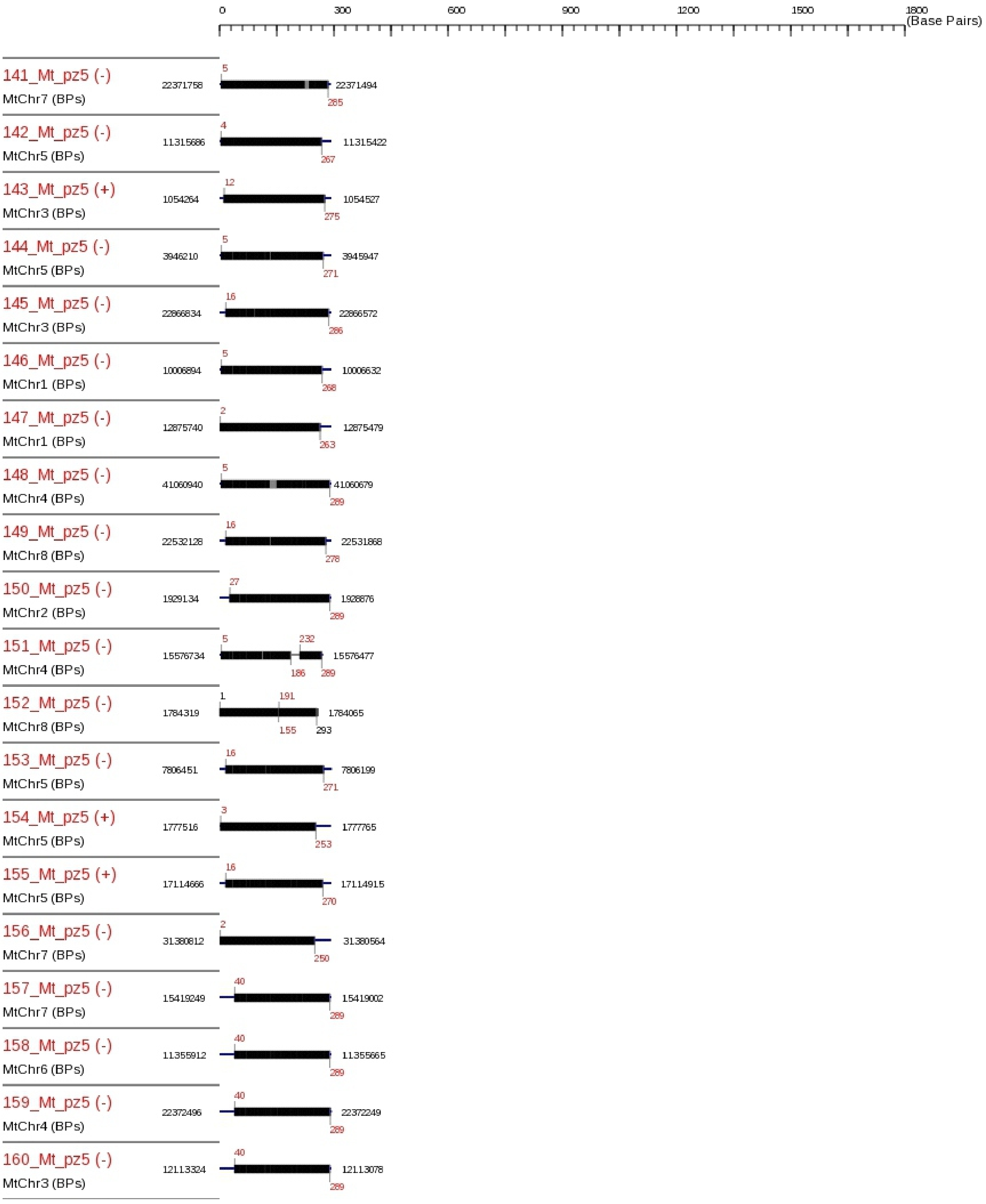

0
300
600
900
1200
1500
1800
(Base Pairs)
141_Mt_pz5 (-)
MtChr7 (BPs)
22371758
5
22371494
285
142_Mt_pz5 (-)
MtChr5 (BPs)
11315686
4
11315422
267
143_Mt_pz5 (+)
MtChr3 (BPs)
1054264
12
1054527
275
144_Mt_pz5 (-)
MtChr5 (BPs)
3946210
5
3945947
271
145_Mt_pz5 (-)
MtChr3 (BPs)
22866834
16
22866572
286
146_Mt_pz5 (-)
MtChr1 (BPs)
10006894
5
10006632
268
147_Mt_pz5 (-)
MtChr1 (BPs)
12875740
2
12875479
263
148_Mt_pz5 (-)
MtChr4 (BPs)
41060940
5
41060679
289
149_Mt_pz5 (-)
MtChr8 (BPs)
22532128
16
22531868
278
150_Mt_pz5 (-)
MtChr2 (BPs)
1929134
27
1928876
289
151_Mt_pz5 (-)
MtChr4 (BPs)
15576734
5
232
186
289
15576477
152_Mt_pz5 (-)
MtChr8 (BPs)
1784319
1
191
155
293
1784065
153_Mt_pz5 (-)
MtChr5 (BPs)
7806451
16
7806199
271
154_Mt_pz5 (+)
MtChr5 (BPs)
1777516
3
1777765
253
155_Mt_pz5 (+)
MtChr5 (BPs)
17114666
16
17114915
270
156_Mt_pz5 (-)
MtChr7 (BPs)
31380812
2
31380564
250
157_Mt_pz5 (-)
MtChr7 (BPs)
15419249
40
15419002
289
158_Mt_pz5 (-)
MtChr6 (BPs)
11355912
40
11355665
289
159_Mt_pz5 (-)
MtChr4 (BPs)
22372496
40
22372249
289
160_Mt_pz5 (-)
MtChr3 (BPs)
12113324
40
12113078
289

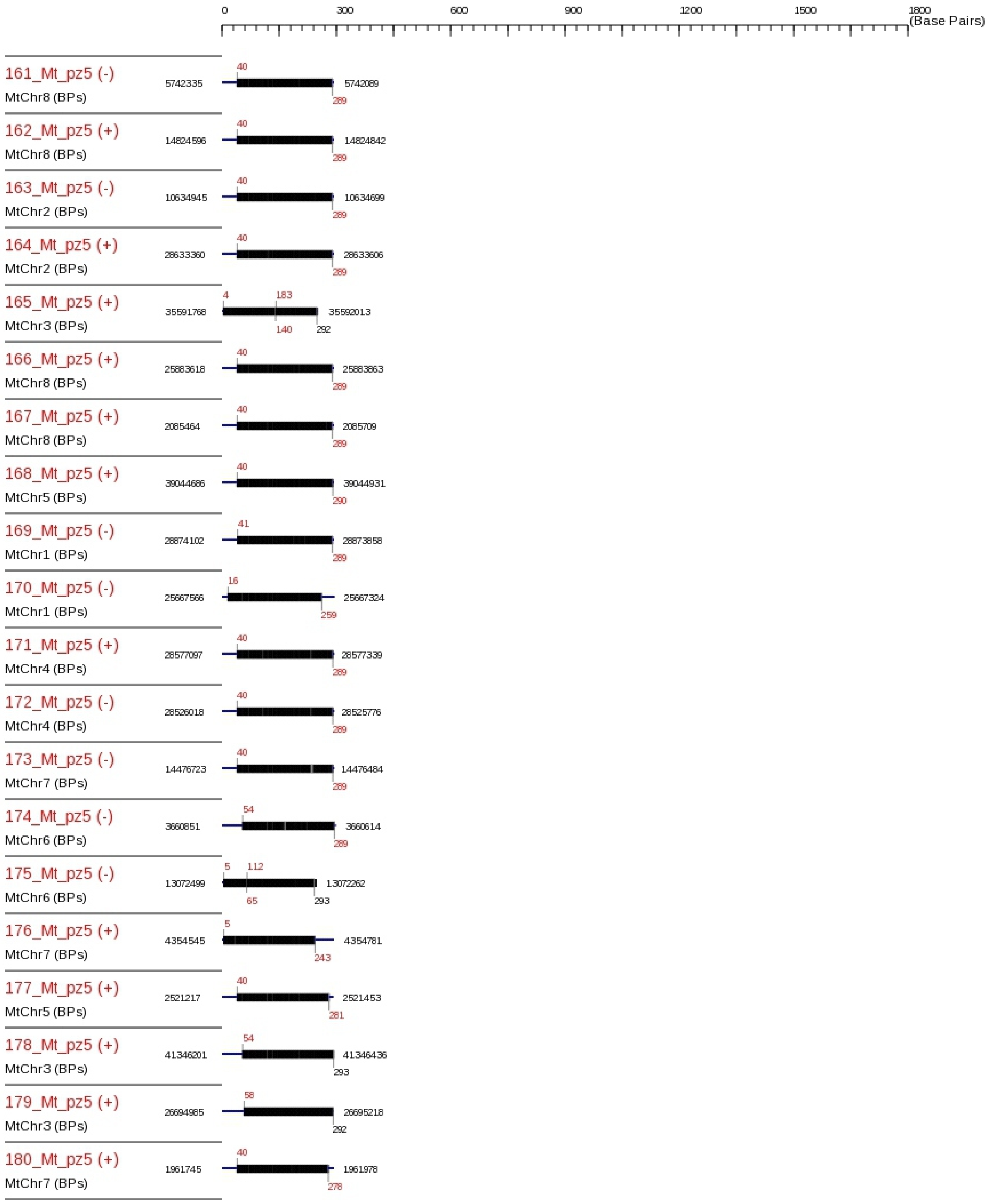
0
300
600
900
1200
1500
1800
(Base Pairs)
161_Mt_pz5 (-)
MtChr8 (BPs)
5742335
40
5742089
289
162_Mt_pz5 (+)
MtChr8 (BPs)
14824596
40
14824842
289
163_Mt_pz5 (-)
MtChr2 (BPs)
10634945
40
10634699
289
164_Mt_pz5 (+)
MtChr2 (BPs)
28633360
40
28633606
289
165_Mt_pz5 (+)
MtChr3 (BPs)
35591768
4
183
35592013
140
292
166_Mt_pz5 (+)
MtChr8 (BPs)
25883618
40
25883863
289
167_Mt_pz5 (+)
MtChr8 (BPs)
2085464
40
2085709
289
168_Mt_pz5 (+)
MtChr5 (BPs)
39044686
40
39044931
290
169_Mt_pz5 (-)
MtChr1 (BPs)
28874102
41
28873858
289
170_Mt_pz5 (-)
MtChr1 (BPs)
25667566
16
25667324
259
171_Mt_pz5 (+)
MtChr4 (BPs)
28577097
40
28577339
289
172_Mt_pz5 (-)
MtChr4 (BPs)
28526018
40
28525776
289
173_Mt_pz5 (-)
MtChr7 (BPs)
14476723
40
14476484
289
174_Mt_pz5 (-)
MtChr6 (BPs)
3660851
54
3660614
289
175_Mt_pz5 (-)
MtChr6 (BPs)
13072499
5
112
13072262
65
293
176_Mt_pz5 (+)
MtChr7 (BPs)
4354545
5
4354781
243
177_Mt_pz5 (+)
MtChr5 (BPs)
2521217
40
2521453
281
178_Mt_pz5 (+)
MtChr3 (BPs)
41346201
54
41346436
293
179_Mt_pz5 (+)
MtChr3 (BPs)
26694985
58
26695218
292
180_Mt_pz5 (+)
MtChr7 (BPs)
1961745
40
1961978
278

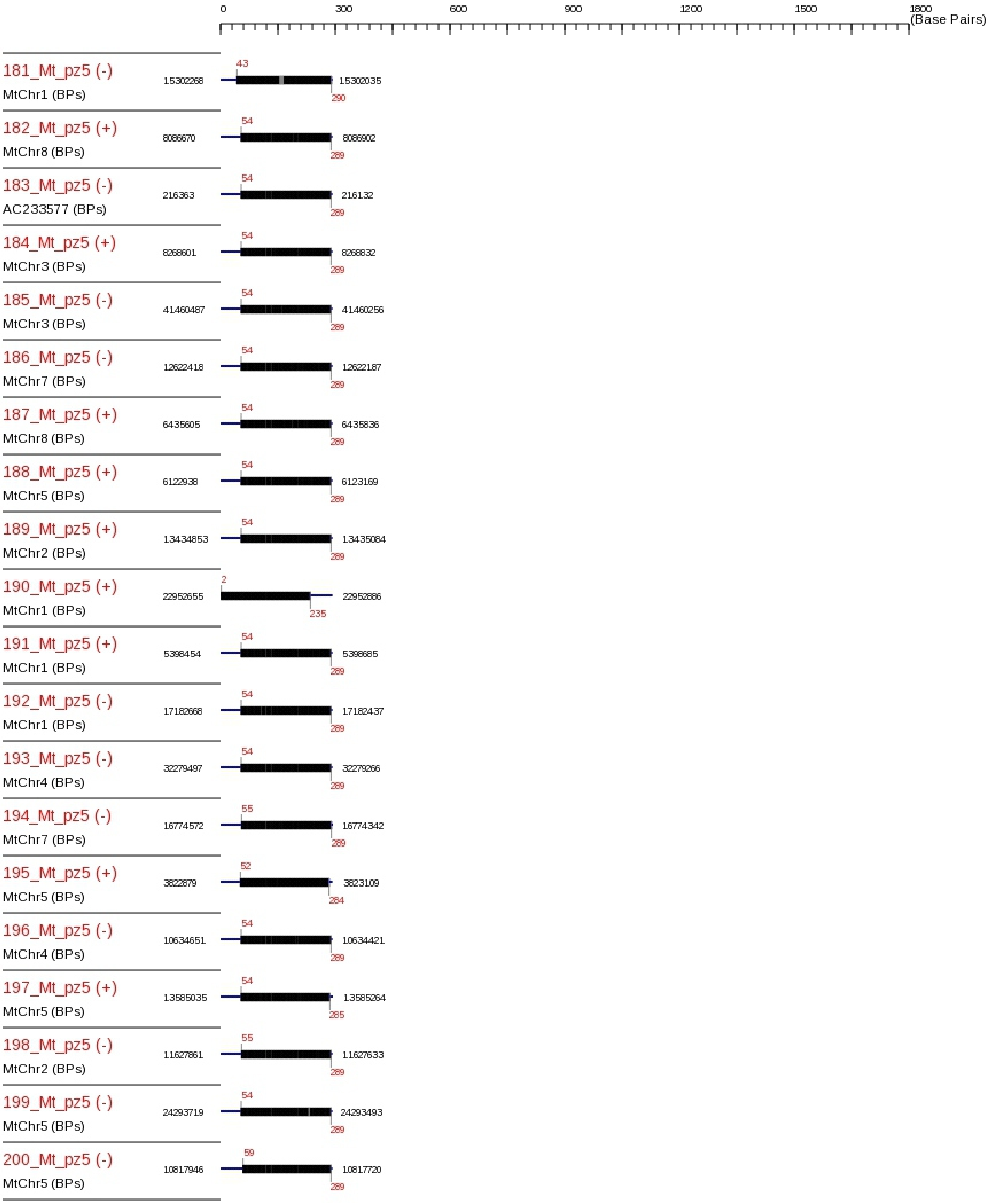

0
300
600
900
1200
1500
1800
(Base Pairs)
181_Mt_pz5 (-)
MtChr1 (BPs)
15302268
43
15302035
290
182_Mt_pz5 (+)
MtChr8 (BPs)
8086670
54
8086902
289
183_Mt_pz5 (-)
AC233577 (BPs)
216363
54
216132
289
184_Mt_pz5 (+)
MtChr3 (BPs)
8268601
54
8268832
289
185_Mt_pz5 (-)
MtChr3 (BPs)
41460487
54
41460256
289
186_Mt_pz5 (-)
MtChr7 (BPs)
12622418
54
12622187
289
187_Mt_pz5 (+)
MtChr8 (BPs)
6435605
54
6435836
289
188_Mt_pz5 (+)
MtChr5 (BPs)
6122938
54
6123169
289
189_Mt_pz5 (+)
MtChr2 (BPs)
13434853
54
13435084
289
190_Mt_pz5 (+)
MtChr1 (BPs)
22952655
2
22952886
235
191_Mt_pz5 (+)
MtChr1 (BPs)
5398454
54
5398685
289
192_Mt_pz5 (-)
MtChr1 (BPs)
17182668
54
17182437
289
193_Mt_pz5 (-)
MtChr4 (BPs)
32279497
54
32279266
289
194_Mt_pz5 (-)
MtChr7 (BPs)
16774572
55
16774342
289
195_Mt_pz5 (+)
MtChr5 (BPs)
3822879
52
3823109
284
196_Mt_pz5 (-)
MtChr4 (BPs)
10634651
54
10634421
289
197_Mt_pz5 (+)
MtChr5 (BPs)
13585035
54
13585264
285
198_Mt_pz5 (-)
MtChr2 (BPs)
11627861
55
11627633
289
199_Mt_pz5 (-)
MtChr5 (BPs)
24293719
54
24293493
289
200_Mt_pz5 (-)
MtChr5 (BPs)
10817946
59
10817720
289

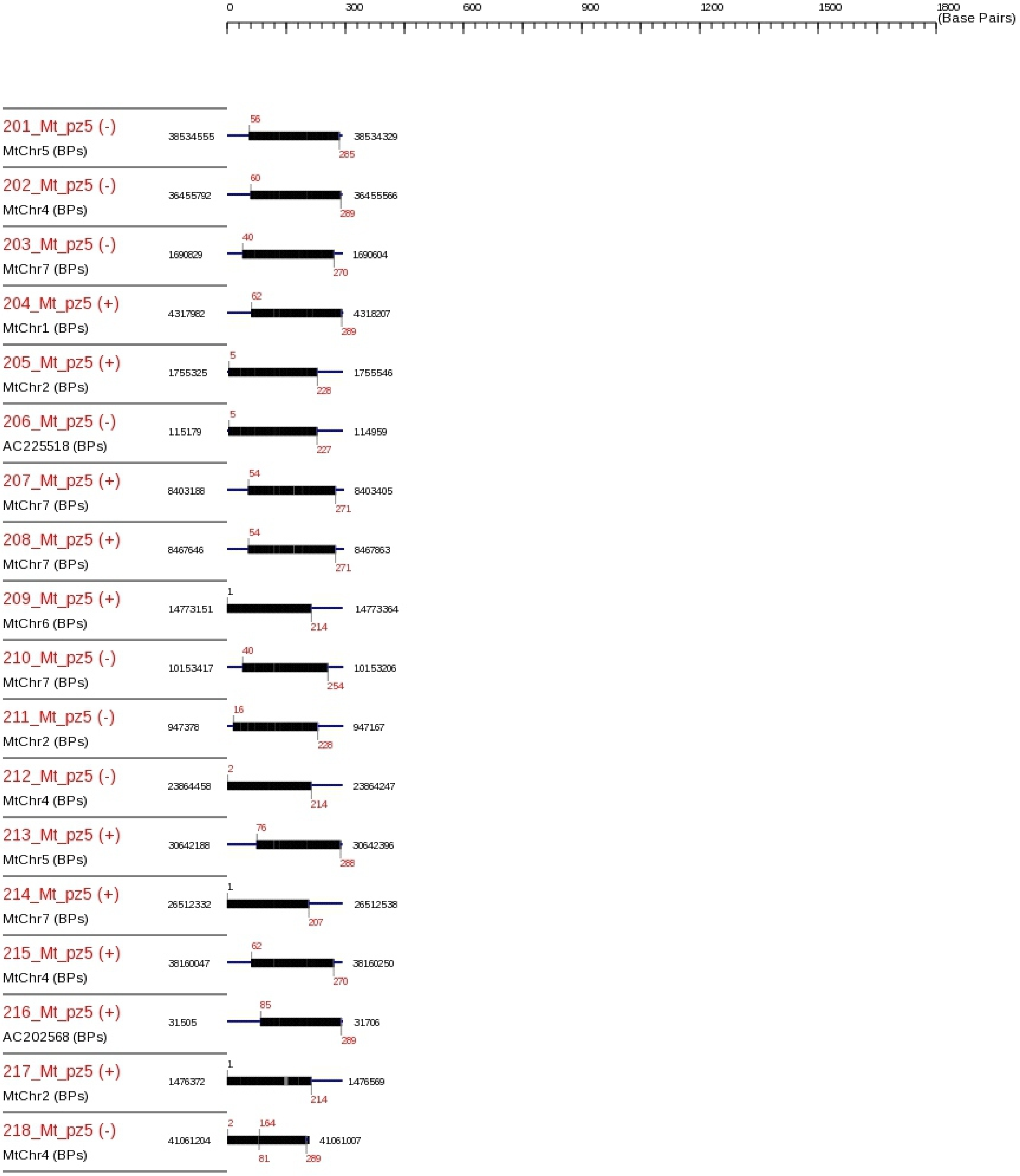

0
300
600
900
1200
1500
1800
(Base Pairs)
201_Mt_pz5 (-)
MtChr5 (BPs)
38534555
56
38534329
285
202_Mt_pz5 (-)
MtChr4 (BPs)
36455792
60
36455566
289
203_Mt_pz5 (-)
MtChr7 (BPs)
1690829
40
1690604
270
204_Mt_pz5 (+)
MtChr1 (BPs)
4317982
62
4318207
289
205_Mt_pz5 (+)
MtChr2 (BPs)
1755325
5
1755546
228
206_Mt_pz5 (-)
AC225518 (BPs)
115179
5
114959
227
207_Mt_pz5 (+)
MtChr7 (BPs)
8403188
54
8403405
271
208_Mt_pz5 (+)
MtChr7 (BPs)
8467646
54
8467863
271
209_Mt_pz5 (+)
MtChr6 (BPs)
14773151
1
14773364
214
210_Mt_pz5 (-)
MtChr7 (BPs)
10153417
40
10153206
254
211_Mt_pz5 (-)
MtChr2 (BPs)
947378
16
947167
228
212_Mt_pz5 (-)
MtChr4 (BPs)
23864458
2
23864247
214
213_Mt_pz5 (+)
MtChr5 (BPs)
30642188
76
30642396
288
214_Mt_pz5 (+)
MtChr7 (BPs)
26512332
1
26512538
207
215_Mt_pz5 (+)
MtChr4 (BPs)
38160047
62
38160250
270
216_Mt_pz5 (+)
AC202568 (BPs)
31505
85
31706
289
217_Mt_pz5 (+)
MtChr2 (BPs)
1476372
1
1476569
214
218_Mt_pz5 (-)
MtChr4 (BPs)
41061204
2
164
41061007
81
289
